# Gel Delivery Systems in Dental Medicine: From Controlled Release to Regenerative Applications

**DOI:** 10.3390/gels11110925

**Published:** 2025-11-19

**Authors:** Dragos Ioan Virvescu, Ionut Luchian, Oana Cioanca, Gabriel Rotundu, Florinel Cosmin Bida, Dana Gabriela Budala, Mihaela Scurtu, Zinovia Surlari, Oana-Maria Butnaru, Monica Hancianu

**Affiliations:** 1 Faculty of Dental Medicine, Grigore T. Popa University of Medicine and Pharmacy, 700115 Iasi, Romania; 2 Faculty of Pharmacy, Grigore T. Popa University of Medicine and Pharmacy, 700115 Iasi, Romania

**Keywords:** gel delivery systems, hydrogels, controlled release, oral drug delivery, regenerative dentistry, nanotechnology

## Abstract

Gel-based delivery systems have emerged as versatile platforms in dentistry due to their biocompatibility, injectability, tunable rheology, and ability to localize therapeutic agents at the site of application. This review synthesizes current evidence on hydrogels, thermosensitive gels, mucoadhesive gels, nanoparticle-loaded gels, and stimuli-responsive systems, highlighting their structural characteristics, mechanisms of drug release, and clinical relevance. Mucoadhesive formulations demonstrate prolonged retention in periodontal pockets and oral mucosa, improving the efficacy of antimicrobials and anti-inflammatory agents. Thermosensitive gels enable minimally invasive administration and in situ gelation, supporting controlled release at body temperature. Nanoparticle-loaded gels exhibit enhanced drug stability and deeper tissue penetration, while “smart” gels respond to environmental stimuli such as pH or temperature to modulate release profiles. Clinical findings indicate reductions in probing depth, improved wound healing, decreased bacterial load, and better patient comfort when gel systems are used as adjuncts to mechanical therapy or regenerative procedures. However, despite these advances, challenges such as variability in gel stability, manufacturing reproducibility, regulatory approval pathways, and limited long-term clinical evidence still constrain widespread adoption of these systems in routine practice.

## 1. Introduction

In recent decades, dentistry has undergone a profound transformation driven by advances in biomaterials, nanotechnology, and minimally invasive therapeutics. Among the most promising developments are gel-based delivery systems, which have opened new perspectives for localized, controlled, and patient-friendly therapies [1]. The oral cavity presents unique clinical and biological challenges-constant exposure to mechanical stress, variable pH, saliva flow, and microbial biofilms—all of which can compromise the effectiveness of conventional treatment method [2]. Consequently, there has been a growing shift toward materials and systems capable of maintaining prolonged contact with oral tissues while delivering therapeutic agents directly to the site of disease [3]. Gel delivery systems have emerged as a central solution within this context.

In the last few years, gel-based delivery methods have become increasingly popular in dentistry since they may be used for both controlled medication release and regenerative purposes.

The oral cavity poses unique challenges for drug administration due to continuous salivary flow, enzymatic activity, variable pH, and mechanical forces, all of which limit the efficacy and residence time of conventional formulations [4,5]. As a result, systemic and topical routes often fail to maintain adequate drug concentrations at the target site, leading to suboptimal therapeutic outcomes and potential systemic side effects [6].

These materials connect old pharmacological methods with new bioengineering ideas. They allow for localized, persistent, and stimuli-responsive release of therapeutic medicines right at the site of disease or tissue injury [7,8].

Initially, gels were introduced primarily as vehicles for antimicrobial and anti-inflammatory agents in the management of periodontal and endodontic infections. Over time, advancements in polymer chemistry, nanotechnology, and tissue engineering have expanded their functionality toward regenerative purposes, including delivery of growth factors, peptides, and stem cells for alveolar bone and periodontal ligament regeneration [9,10].

Despite extensive progress in material science and drug delivery research, the application of gel-based systems in dentistry remains fragmented across disciplines and therapeutic areas. Most published studies have focused on formulation development or isolated clinical uses, often lacking an integrated perspective that connects physicochemical characteristics with biological performance and clinical outcomes.

Compared with other local delivery platforms—such as bioresorbable films, microspheres, and nanocarriers—gel-based systems address several unmet clinical challenges in dentistry. Films often lack conformability in deep or irregular periodontal pockets and may detach under salivary flow. Microspheres and nanocarriers can provide controlled release, but their retention is limited without a supportive matrix or adhesive carrier. In contrast, gels offer injectable or moldable formats that adapt to the tissue surface, maintain close contact with the target site, and can be formulated to exhibit mucoadhesion and in situ gelation.

These characteristics are particularly advantageous in periodontal therapy, mucosal wound management, and regenerative procedures, where localized, sustained delivery and mechanical stability against dynamic oral forces are essential.

However, most existing reports address these systems in a descriptive manner, without critically comparing their performance, limitations, and clinical readiness. To address this, the present review does not only summarize gel delivery systems but also examines their functional trade-offs, stability challenges, and clinical evidence strength. Where possible, we highlight limitations in manufacturing reproducibility, retention under salivary flow, variability in drug release kinetics, and the scarcity of long-term randomized trials. By doing so, this review aims to provide a more analytical perspective, identifying which gel types are currently suitable for clinical use and where future research is still required.

This review provides a general overview of gel delivery systems in dentistry, emphasizing their evolution from simple controlled-release formulations toward multifunctional platforms with regenerative potential. It aims to outline the conceptual advances that have shaped their clinical relevance and to highlight the growing role of these systems in achieving more effective, predictable, and biologically integrated dental treatments.

## 2. Literature Review

The evolution of gel delivery systems reflects the progressive adaptation of pharmaceutical principles to the specific conditions of the oral environment. From simple viscous carriers used for local drug application to sophisticated polymeric matrices capable of controlled release, their development has paralleled advances in material science and clinical needs. Understanding this historical trajectory provides context for the current diversity of gel formulations used in dentistry.

### 2.1. Historical Evolution and Classification of Gel Delivery Systems

The pharmaceutical sciences were the first to develop viscous semi-solid formulations to aid diluent medication retention on mucosal surfaces, which led to the concept of using gels as therapeutic carriers [11]. Simple aqueous or polymeric bases held antiseptics, fluoride agents, or anti-inflammatory drugs at the commencement of dental procedures [12]. Over time, advancements in biomaterials, polymer chemistry, and nanotechnology transformed gels from inert carriers into active systems that could regulate drug release, increase drug availability in the body, and facilitate tissue regeneration [13].

In dentistry, this evolution was driven by the need to overcome the challenges of the oral environment—constant saliva flow, enzymatic degradation, variable pH, and mechanical stress—which limit the efficacy of traditional dosage forms [14]. As a result, gel systems have been increasingly engineered to ensure longer residence time, improved adhesion to oral tissues, and sustained therapeutic activity.

This historical progression reflects a shift from simple viscous carriers toward engineered polymeric systems with increasingly controlled and adaptive release characteristics. While early gels primarily served to prolong surface contact, modern systems incorporate structural modifications that enhance stability, retention, and therapeutic precision. Currently, the development of gel-based delivery systems is guided by the need to balance biocompatibility, mechanical resilience under salivary flow, and predictable release kinetics [15,16], as seen in Table 1.

### 2.2. Transition from Conventional to Smart Gel Systems

Initially, gels in dentistry functioned mainly as passive carriers designed to extend local drug contact. Advances in polymer chemistry have progressively introduced systems capable of responding to environmental conditions such as temperature, pH, or enzymatic activity. These “smart” gels enable targeted and on-demand release, improving therapeutic efficiency while reducing systemic exposure. This evolution marks a transition from viscosity-dependent retention toward dynamic and biointeractive delivery platforms [17].

Modern “smart” gels can adjust their behavior according to environmental stimuli such as temperature, pH, ionic strength, or enzymatic activity, enabling on-demand and site-specific drug release [18]. These systems integrate dynamic molecular networks capable of undergoing sol–gel transitions or controlled swelling in response to oral conditions, thus maintaining optimal therapeutic concentrations for longer durations [19,20].

Another recent study reported the development of a thermosensitive and mucoadhesive gel containing solid lipid nanoparticles loaded with fluconazole and niosomes loaded with clindamycin, designed for the local treatment of periodontal disease. The formulation demonstrated prolonged drug release and enhanced antimicrobial activity compared to free suspensions [21].

Moreover, bioactive thermo-responsive hydrogels incorporating growth factors or bio-functional peptides have shown promise in promoting periodontal and alveolar tissue healing by combining controlled drug delivery with regenerative potential [22]. Altogether, these advances highlight the ongoing shift from conventional, viscosity-dependent formulations toward adaptive gel-based biomaterials designed for controlled drug release, responsiveness to specific oral stimuli, and active interaction with surrounding tissues. This evolution reflects a move toward intelligent, bio-interactive delivery systems, capable of integrating with the biological environment to achieve more predictable, personalized, and outcome-driven dental therapies [23].

### 2.3. Types of Gel-Based Systems Used in Dentistry

Gel systems used in dentistry vary in composition, responsiveness, and clinical purpose. Their differences can be broadly understood by distinguishing structural hydrogels (which provide a hydrated matrix with controllable degradation) from functional systems that incorporate mucoadhesive, thermosensitive, or nanoparticle-mediated mechanisms to enhance retention, delivery precision, or regenerative support. In dentistry, four major categories of gel systems have gained clinical and research relevance: hydrogels and thermosensitive gels, mucoadhesive gels, nanoparticle-loaded gels, and stimuli-responsive gels.

#### 2.3.1. Hydrogels and Thermosensitive Gels

Hydrogels are three-dimensional, hydrophilic polymeric networks capable of absorbing large quantities of water while maintaining structural integrity. Their similarity to natural extracellular matrix makes them particularly suitable for oral applications, such as controlled release of antimicrobials, anti-inflammatories, or growth factors in periodontal and endodontic therapy [24].

**Figure 1:**
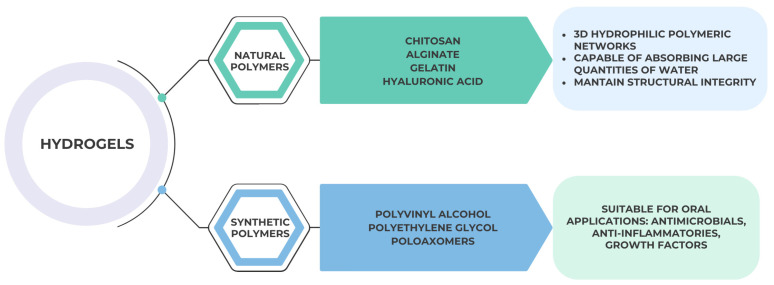
Structural representation and classification of hydrogels used in dentistry, illustrating their three-dimensional hydrophilic polymeric network, composition (natural vs. synthetic polymers), and key properties relevant to controlled drug delivery and biocompatibility.

**Figure 2:**
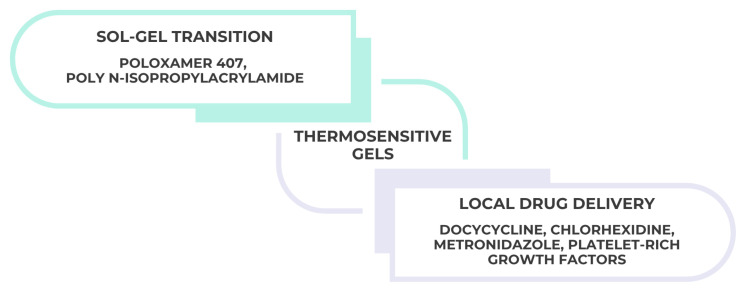
Schematic representation of thermosensitive gel systems used in dentistry. Poloxamer 407 (Pluronic^®^ F127) and poly(N-isopropylacrylamide) undergo a sol–gel transition when temperature rises from ambient to body conditions, enabling minimally invasive administration and prolonged retention at periodontal or mucosal sites for localized drug delivery.

They can be based on natural polymers (e.g., chitosan, alginate, gelatin, hyaluronic acid) or synthetic polymers (e.g., polyvinyl alcohol, polyethylene glycol, poloxamers). The choice of polymer determines swelling behavior, degradation rate, and release kinetics as shown in Figure 1:

Thermosensitive gels represent a special subclass that undergo sol–gel transition in response to temperature. Systems containing Poloxamer 407 (Pluronic^®^ F127) or poly(N-isopropylacrylamide) remain liquid at room temperature and transform into semi-solid gels at body temperature, facilitating minimally invasive administration and prolonged retention in the periodontal pocket or mucosal surface [25,26]. These gels improve drug bioavailability and reduce systemic exposure, and have been successfully applied for local delivery of doxycycline, chlorhexidine, metronidazole, and platelet-rich growth factors, as illustrated in Figure 2 below:

#### 2.3.2. Mucoadhesive Gels

**Figure 3:**
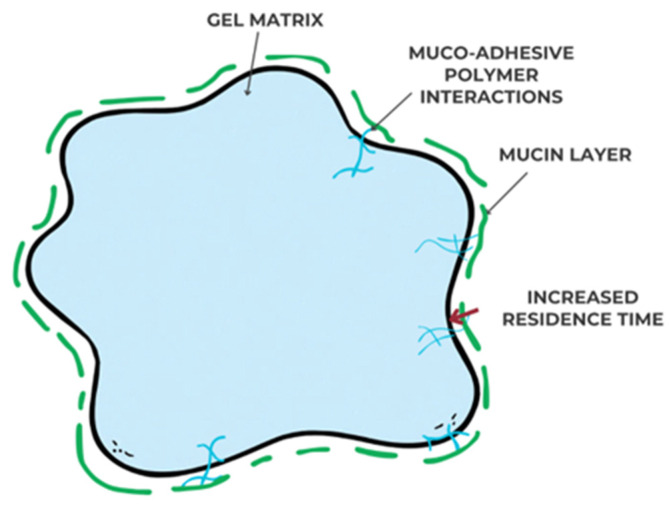
Schematic representation of mucoadhesive interactions between the polymeric gel network and the mucin layer.

Mucoadhesive gels are designed to adhere to the oral mucosa or gingival tissues through hydrogen bonding, electrostatic interactions, or physical entanglement with mucin glycoproteins. The mucoadhesive property enhances the residence time of the formulation at the target site, protecting the drug from salivary dilution and enzymatic degradation [27], as seen in Figure 3 below:

Common mucoadhesive polymers include carbopol, hydroxypropyl methylcellulose (HPMC), sodium carboxymethylcellulose, and chitosan, alone or in combination with plasticizers and cross-linkers. These gels are particularly advantageous in managing periodontal pockets, aphthous ulcers, and post-surgical wounds, where sustained topical exposure improves healing and antimicrobial efficiency [28].

Clinical studies have demonstrated significant improvements in pocket depth reduction and bacterial load when mucoadhesive chlorhexidine or doxycycline gels were used as adjuncts to scale and root planning [29,30].

#### 2.3.3. Nanoparticle-Loaded Gels

The incorporation of nanoparticles (NPs) into gel matrices has emerged as a key strategy for enhancing drug stability, tissue penetration, and controlled release profiles. Nanoparticle-loaded gels (NP-gels) combine the structural advantages of gels with the nanoscale delivery precision of particles such as liposomes, polymeric nanoparticles, solid lipid nanoparticles, or metallic nanocarriers [31].

In periodontal and endodontic therapy, chitosan- or PLGA (Polylactic-co-glycolic acid)-based nanoparticle gels have been developed for sustained delivery of antibiotics (metronidazole, ciprofloxacin), anti-inflammatory agents, and regenerative biomolecules such as BMP-2(Bone Morphogenetic Protein-2) and VEGF [32].

**Figure 4:**
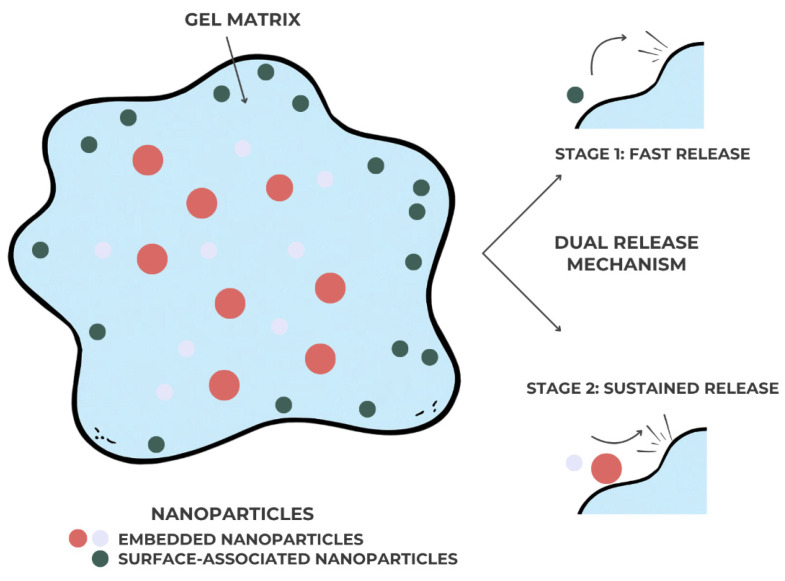
Schematic representation of nanoparticle-loaded gels used in dental applications.

The gel matrix stabilizes nanoparticles and provides a dual release mechanism—initial diffusion from the gel and subsequent gradual release from the nanoparticles—allowing effective concentrations at the target site over extended periods. Additionally, metallic nanoparticles (e.g., Ag, ZnO, TiO_2_) confer intrinsic antimicrobial and antibiofilm activity, enhancing the therapeutic effect [33], as seen in Figure 4:

Comparative evidence indicates that the therapeutic efficacy of nanoparticle-loaded hydrogels varies depending on the incorporated agent. Bioactive glass nanoparticles promote mineral deposition and support periodontal regeneration by stimulating fibroblast proliferation and enhancing early osteogenic signaling. In contrast, zinc oxide nanoparticles primarily contribute potent antimicrobial activity through the release of Zn^2+^ ions, disrupting bacterial cell membranes and biofilm architecture, with additional anti-inflammatory effects reported in gingival tissues. Meanwhile, chlorhexidine-loaded hydrogels are mainly associated with sustained local antimicrobial delivery, leading to reduced pocket depth and bacterial load when used adjunctively to scaling and root planing.

While bioactive glass demonstrates superior regenerative potential and zinc oxide provides a broader antibacterial spectrum, chlorhexidine hydrogels remain the most clinically validated option to date due to their established safety and predictable therapeutic performance.

#### 2.3.4. Stimuli-Responsive (“Smart”) Gels

The latest evolution in dental gel systems includes stimuli-responsive or “smart” gels, which can reversibly modify their properties in response to environmental cues such as pH, temperature, ionic strength, or enzymatic activity [34]. These systems provide on-demand drug release synchronized with pathological conditions—such as infection, inflammation, or acidic pH in periodontal pockets.

For example, pH-sensitive chitosan–polyacrylic acid gels release antibiotics preferentially in acidic microenvironments characteristic of infection sites. Similarly, enzyme-degradable hydrogels cross-linked with matrix metalloproteinase (MMP)-sensitive peptides degrade in proportion to inflammatory enzyme activity, offering precise temporal control of drug delivery [35,36].

Smart or stimuli-responsive gels are designed to alter their structural organization or drug release behavior in response to specific environmental triggers. These triggers may be physical, such as temperature or pH changes, or chemical, such as enzyme activity or ionic composition at the disease site. For example, thermosensitive systems undergo sol–gel transition when temperature increases from ambient to physiological conditions, improving in situ retention in periodontal pockets [37]. In contrast, pH-responsive gels modulate drug release under acidic conditions associated with inflammation or infection, ensuring targeted antimicrobial delivery.

Additionally, enzyme-degradable hydrogels cross-linked via matrix metalloproteinase (MMP)-sensitive peptides have demonstrated controlled degradation correlated with inflammatory enzyme levels, supporting regenerative procedures in periodontal defects [37].

**Figure 5:**
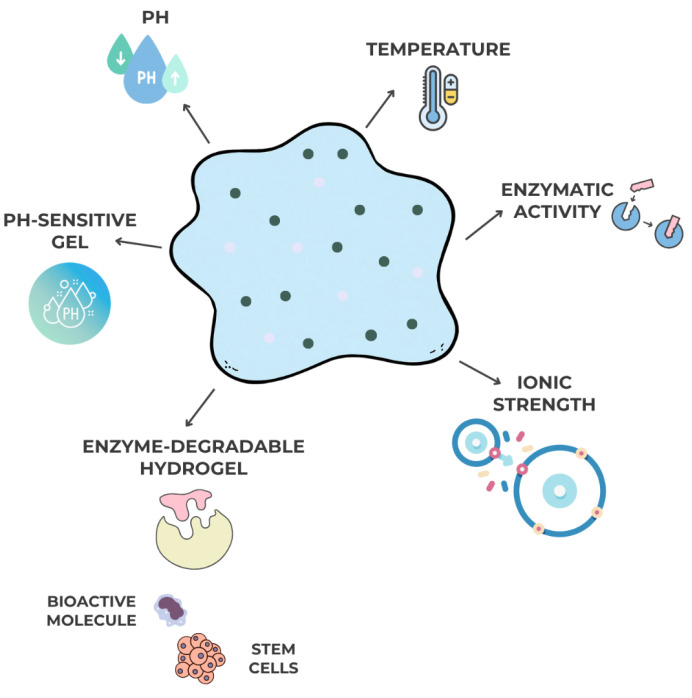
Stimuli-responsive (“smart”) gels in dentistry: schematic representation of pH-, temperature-, and enzyme-sensitive hydrogel systems enabling on-demand drug release and regenerative responses in periodontal therapy and tissue engineering applications.

Smart systems are also being engineered to carry bioactive molecules and stem cells, opening new perspectives in periodontal tissue engineering and regenerative endodontics, where responsiveness to the healing microenvironment is essential [38], as in Figure 5 below:

Across these gel systems, it is important to note that their advantages are often counterbalanced by clinically relevant limitations. Hydrogels provide biocompatibility but may lack sufficient retention under dynamic oral conditions. Thermosensitive gels offer convenience in situ gelation yet can show batch-to-batch variability in gelation temperature and mechanical stability. Mucoadhesive gels improve residence time, but their performance is highly dependent on local salivary flow and mucosal integrity. Nanoparticle-loaded gels achieve deeper penetration and controlled release, but their regulatory and long-term safety profiles remain insufficiently established. These trade-offs highlight the need for careful system selection based on the specific anatomical site, therapeutic target, and expected duration of action.

### 2.4. Physicochemical and Biological Properties

The performance of gel-based systems in dentistry depends on a complex interplay between their physicochemical and biological characteristics. The formulation parameters—polymer type, concentration, cross-linking density, and solvent composition—govern the mechanical behavior, mucoadhesive capacity, degradation rate, and overall biocompatibility. Understanding these interrelated properties is crucial for designing gels capable of ensuring controlled drug release, patient comfort, and predictable therapeutic outcomes in the dynamic oral environment [39].

#### 2.4.1. Rheology, Gelation, and Viscosity

Rheological behavior defines the viscoelastic nature and flow properties of gels, determining their injectability, spreadability, and residence time at the target site. Ideal dental gels exhibit pseudoplastic or shear-thinning behavior, allowing easy application under mechanical stress and rapid recovery of viscosity afterward [40,41].

The gelation process—transition from sol to gel—can occur through temperature changes, ionic cross-linking, or polymer entanglement. For example, thermosensitive gels containing Poloxamer 407 or PNIPAAm (Poly(N-isopropylacrylamide) exhibit sol–gel transition near physiological temperature, optimizing ease of administration and in situ retention [42].

Viscosity directly influences the release rate of the drug: highly viscous formulations slow diffusion but increase retention in the periodontal pocket or mucosal surface. Achieving an optimal balance between mechanical stability and drug mobility remains a formulation challenge in oral gels [41].

#### 2.4.2. Mucoadhesion, Degradation, and Biocompatibility

Mucoadhesion is a fundamental property that determines the capacity of polymeric gels to adhere to the oral mucosa, ensuring prolonged residence time, localized drug release, and improved bioavailability. The process occurs in two main stages: an initial wetting and swelling phase, in which the hydrated polymer spreads over the mucosal surface, followed by interpenetration and bonding between the polymer chains and mucin glycoproteins [43].

It is important to note that muco-adhesion values measured in vitro do not directly translate to vivo performance. In vitro tests are typically conducted under static or semi-static conditions, whereas the oral cavity is a highly dynamic environment characterized by continuous salivary flow, mechanical shear from mastication, and enzymatic turnover of mucus glycoproteins.

High muco-adhesion force in vitro may therefore not guarantee prolonged retention if the formulation exhibits rapid hydration, erosion, or limited resistance to shear stress. Conversely, systems with moderate in vitro muco-adhesion may perform better in vivo if they possess viscoelasticity and swelling behavior that allow adaptation to tissue microtopography. For these reasons, mucoadhesive performance must be interpreted in the context of both polymer–mucin interactions and the gel’s rheological stability under physiological conditions. Therefore, in vivo evaluation should include parameters such as retention duration under simulated salivary flow, enzymatic degradation rate, and resistance to mechanical displacement, which more accurately reflect clinical conditions.

In gel-based drug delivery systems, muco-adhesion represents a critical determinant of therapeutic performance, governing both residence time and local drug bioavailability within the oral cavity [44].

When incorporated into hydrated polymeric gels, mucoadhesive components establish non-covalent interfacial bonds such as hydrogen, ionic, and van der Waals interactions [45] with the mucosal layer, enabling prolonged retention under dynamic physiological conditions. This interfacial behavior depends on the viscoelastic properties of the gel matrix and on its ability to absorb water, swell, and interact with mucin glycoproteins at the epithelial surface [46].

Degradation behavior plays a decisive role in controlling the structural stability, drug release kinetics, and biocompatibility of gel-based delivery systems. Biodegradable gels are designed to gradually disintegrate within the physiological environment through enzymatic or hydrolytic cleavage of their polymeric backbone, yielding non-toxic, easily resorbable by-products [47].

In enzymatic degradation, specific enzymes present in saliva or gingival crevicular fluid catalyze the cleavage of glycosidic or ester bonds within the polymer matrix. This process is particularly relevant for natural polymers like chitosan, gelatin, and hyaluronic acid, whose degradation rate depends on enzyme concentration, molecular weight, and degree of deacetylation or crosslinking [48].

On the other side, hydrolytic degradation happens when water molecules spontaneously interact with connections that are susceptible to hydrolysis, including esters, amides, or anhydrides. When water diffuses into the gel network, it starts cleavage and mass loss, which is the most common mechanism in synthetic polyesters (e.g., PLGA, PCL, or PEG-based copolymers). The hydrolysis rate is influenced by polymer hydrophilicity, crystallinity, and pH, determining whether the erosion follows a surface-controlled or bulk-controlled pattern [49].

Biocompatibility is essential for oral formulations that remain in prolonged contact with gingival or mucosal tissues. Cytotoxicity, irritation, or immune reactions can occur if residual monomers, surfactants, or cross-linkers leach from the gel [50]. Therefore, formulations must comply with ISO 10993 and ISO 7405 biocompatibility standards for dental biomaterials [51,52].

### 2.5. Mechanisms of Drug Release

Diffusion, swelling, and degradation control the release of drugs from gel matrices in the oral cavity, which in turn determines the formulation’s therapeutic profile and residence time frame [53]. The drug molecules passively migrate across the hydrated polymeric network, which is regulated by the diffusion mechanism [54]. The drug molecular weight, crosslinking density, and mesh size of the polymer determine this process mainly. Polymer degradation slowly erodes the structure, allowing for sequential or sustained release, in swelling or erosion-controlled systems. Encapsulated pharmaceuticals are transported more easily as the gel matrix expands due to water uptake [55,56].

Importantly, drug release behavior is also influenced by the intrinsic physicochemical properties of the active compound itself, not solely by the hydrogel matrix. Factors such as molecular weight, charge, hydrophobicity, and especially water solubility determine the drug’s diffusion coefficient and its affinity to the polymer network. Hydrophilic and low-molecular-weight drugs generally diffuse more rapidly through the hydrated gel structure, whereas poorly water-soluble or hydrophobic agents tend to show slower, more sustained release due to stronger interactions and reduced mobility within the gel matrix.

Another cutting-edge type of gel is the stimuli-responsive gel, which synchronizes the release rate with pathological circumstances in the mouth by adjusting drug liberation in response to local environmental cues like changes in temperature, ionic strength, pH, or enzyme activity [57].

**Figure 6:**
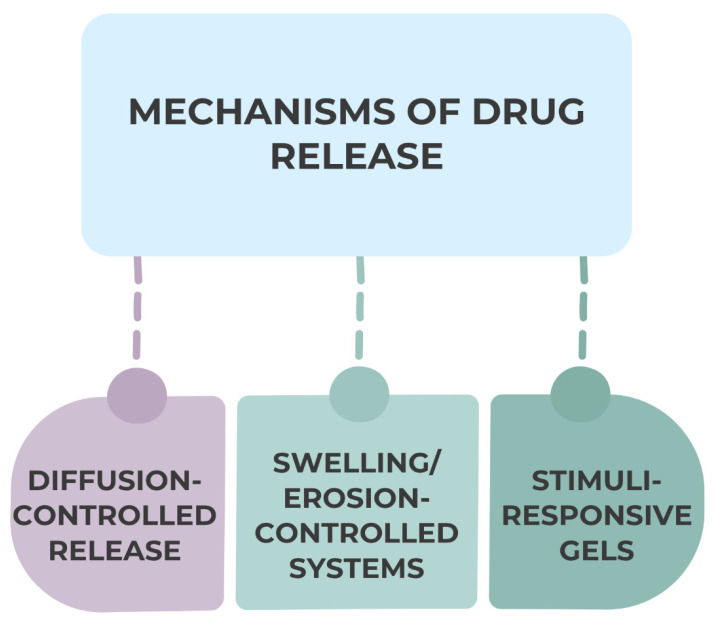
Mechanisms of drug release from gel matrices in the oral cavity.

**Figure 7:**
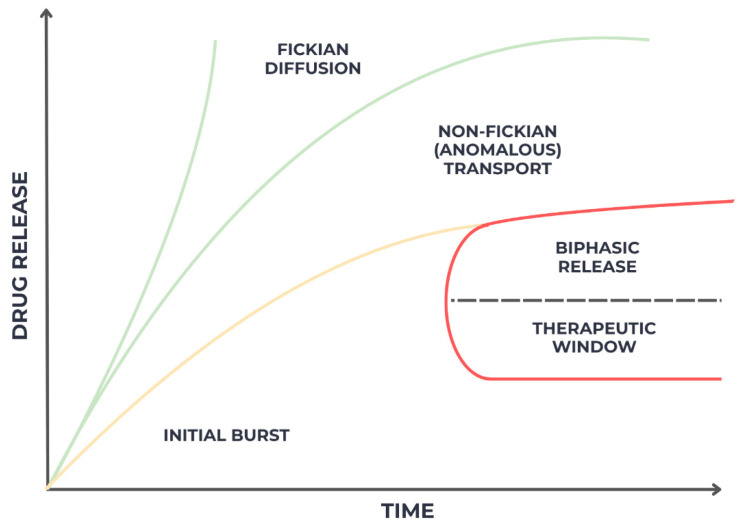
Mathematical modeling of drug release from gel matrices.

The dynamic oral environment introduces further complexity: fluctuating hydration levels, salivary flow variations, and continuous mechanical stimulation from mastication and speech can modify gel microstructure and diffusion pathways [58]. Enzymes present in saliva may also catalyze partial degradation of the polymer backbone, altering drug mobility and kinetics. These interactions, summarized in Figure 6, highlight the intricate balance between gel composition, drug properties, and oral physiological conditions that ultimately define the efficiency and predictability of controlled release systems in dentistry:

Mathematical modeling plays a crucial role in understanding and optimizing the release behavior of therapeutic agents from gel-based systems. Kinetic models such as Higuchi’s square-root law, Korsmeyer–Peppas, and first-order release equations are frequently applied to describe drug diffusion dynamics and to differentiate between Fickian (diffusion-controlled) and non-Fickian (anomalous or swelling-controlled) transport processes as seen in Figure 7 [59]. These models enable the prediction of release kinetics under physiological conditions and guide the rational design of gel formulations with desired therapeutic profiles.

Ideally, optimized formulations exhibit a biphasic release pattern—an initial burst phase that provides rapid antimicrobial or anti-inflammatory activity, followed by a sustained release phase that maintains drug concentration within the therapeutic window over an extended period. Such controlled kinetics are particularly advantageous in oral environments, where constant salivary flow and mechanical forces can otherwise compromise drug retention and efficacy [60].

In practice, these mechanisms do not occur in isolation. Depending on the polymer network architecture, cross-linking density, and environmental conditions, diffusion, swelling, and degradation may act sequentially or simultaneously. For example, highly hydrated and loosely cross-linked gels may permit rapid diffusion as the primary release mechanism, while more structured hydrogels may require matrix swelling or partial degradation before drug molecules are able to migrate. Therefore, the dominant release pathway is formulation dependent and may shift over time during in situ gel residence.

### 2.6. Clinical Applications

Over time, gel-based systems have progressed from being basic drug delivery vehicles to being complex bioactive platforms that can facilitate regeneration and healing. Periodontology, endodontics, oral surgery, and mucosal therapy are just a few of the dental specialties that can benefit from their biocompatibility, variable viscosity, and sustained release of therapeutic ingredients.

#### 2.6.1. Periodontal Therapy

One of the earliest and most established uses of gels in dentistry is the local delivery of antimicrobials within periodontal pockets [61].

Conventional mechanical debridement through scaling and root planing (SRP) remains the gold standard for nonsurgical therapy; however, its efficacy is limited in deep or complex pockets where residual bacterial biofilms persist. In this context, locally applied gel-based formulations have emerged as valuable adjuncts capable of sustaining therapeutic drug concentrations directly at the disease site while minimizing systemic exposure.

The therapeutic results of formulations combining SRP with doxycycline, metronidazole, chlorhexidine, or minocycline incorporated in hydrogel or thermosensitive matrix have been excellent. Improvements in clinical attachment levels, decreased probing depth, bleeding during probing, and microbiological load were observed in multiple randomized controlled trials when compared to SRP alone [62,63,64]. Effectively breaking biofilm formation and reducing recolonization, the localized release from gel carriers guarantees extended exposure of subgingival bacteria to antimicrobials.

**Figure 8:**
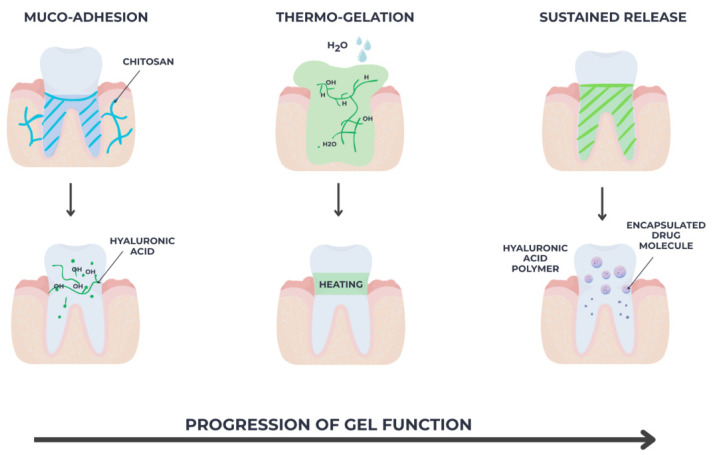
Progressive functional enhancement of the gel system: muco-adhesion, thermo-gelation and sustained release achieved by embedding nanoparticles within the polymeric matrix.

To enhance bio-adhesion to gingival tissues and regulate drug release rate, newer systems incorporate bio-responsive polymers such chitosan, poloxamer, hyaluronic acid, or polyethylene glycol, or nanocarriers [65,66]. Chitosan-based hydrogels, for example, provide intrinsic antimicrobial and wound-healing properties, while hyaluronic acid enhances tissue hydration and epithelial regeneration. Additionally, poloxamer-based thermosensitive gels remain liquid at room temperature and solidify at body temperature, facilitating minimally invasive application and prolonged retention in the periodontal pocket, as seen in Figure 8 below [67,68]:

Beyond antimicrobial delivery, biologically active gels such as platelet-rich fibrin (PRF), platelet gel, and growth factor–enriched matrices have gained increasing attention for their regenerative potential. These formulations not only accelerate soft-tissue repair and angiogenesis but also promote alveolar bone fill in intrabony defects through the sustained release of autologous cytokines and growth factors, including PDGF, VEGF, and TGF-β [67]. Their biocompatibility and patient-derived origin make them particularly appealing for personalized regenerative therapies that bridge the gap between conventional pharmacologic gels and true tissue-engineering approaches.

Although improvements in probing depth and clinical attachment level have been reported, the magnitude and durability of these effects vary substantially across studies. Many clinical trials include small sample sizes and short follow-up periods, and few directly compare gel types under standardized periodontal conditions. As such, current evidence supports their use as adjuncts but does not yet establish consistent superiority of one gel system over another in long-term periodontal maintenance.

#### 2.6.2. Endodontic Applications

In endodontics, gels are used to deliver antimicrobial and regenerative agents into the root canal system. Calcium hydroxide gels remain a cornerstone of intracanal disinfection, while newer thermo-responsive and nanoparticle-enriched hydrogels allow sustained release of antibiotics and growth factors [68,69].

Incorporation of bioactive glass nanoparticles, zinc oxide, or chlorhexidine in hydrogel matrices improves antimicrobial performance against *Enterococcus faecalis* and biofilm-forming species [70,71]. Hydrogel scaffolds have recently come to the center of regenerative endodontic therapy due to their critical function as bioactive carriers of growth factors and stem cells. Hydrogels allow for biological regeneration instead of just obturation by creating a hydrated three-dimensional microenvironment that promotes cell adhesion, migration, and differentiation inside the pulp space [72].

Several in vitro and in vivo studies have demonstrated that incorporating signaling molecules such as bone morphogenetic protein-2 and vascular endothelial growth factor into hydrogels significantly enhances pulp–dentin complex regeneration and neovascularization. For example, Elnawam et al. developed a bovine dental pulp–derived extracellular matrix hydrogel enriched with BMP-2, TGF-β1, and VEGF, showing improved odontoblastic differentiation and angiogenic potential in vitro, suggesting strong potential for clinical translation in regenerative endodontics [73].

Similarly, Xie et al. designed a biomimetic gelatin–methacrylate (GelMA) hydrogel mimicking the native pulp ECM, which, when loaded with VEGF, promoted vascular network formation and dentin bridge deposition in rat molars, confirming the angiogenic and regenerative benefits of VEGF-based hydrogel systems [74].

Shamszadeh et al. highlighted in their recent systematic review that hydrogel-based growth factor delivery, especially of BMP-2, VEFG, and FGF-2, improves cell homing and mineralization of the dentin-pulp interface, surpassing traditional methods based on blood clots in preclinical models [75].

Hydrogels offer a biomimetic microenvironment that closely resembles the native extracellular matrix (ECM), enabling efficient adhesion, migration, and lineage differentiation of dental pulp stem cells (DPSCs). Their high-water content and viscoelasticity permit cell movement through the matrix, unlike conventional rigid scaffolds that may restrict cellular spreading or require enzymatic remodeling before migration can occur.

Additionally, hydrogels allow for the incorporation of bioactive molecules (e.g., RGD peptides, growth factors such as BMP-2, PDGF, or VEGF), which provide biochemical cues that direct odontogenic and angiogenic differentiation.

The nanoscale porosity and tunable cross-linking density further support dynamic cell–matrix interactions, enhancing cytoskeletal organization and signaling pathways such as MAPK/ERK and Wnt/β-catenin, which are associated with reparative dentin formation. In contrast, traditional scaffolds—typically more rigid and structurally static—may facilitate cell anchorage but often lack the adaptive mechanical compliance and molecular signaling environment required for optimal DPSC migration and dentin–pulp complex regeneration.

Collectively, these findings support the paradigm shift toward bioactive, growth factor–enriched hydrogels that combine stem cell therapy and controlled molecular signaling to reestablish the vascularized pulp–dentin complex. Such strategies move beyond passive root canal filling to truly biological regeneration, integrating cell biology, material science, and molecular signaling into a cohesive therapeutic framework.

Despite promising biological outcomes in vitro and in animal models, translation to predictable human clinical protocols remains limited. Standardized outcome measures, long-term follow-up, and multicenter clinical trials are needed to determine whether these regenerative gel-based systems can reliably outperform conventional endodontic therapies.

#### 2.6.3. Oral Surgery and Wound Management

Following surgical interventions such as tooth extractions, implant placement, or soft tissue grafting, bio-adhesive gels play a key role in post-operative management by maintaining a moist healing environment, reducing bacterial contamination, and modulating the inflammatory response. Their viscoelastic nature allows close adaptation to the wound surface, prolonging contact time and enhancing therapeutic efficacy [76]. A recent systematic review/meta-analysis on third-molar surgery concluded that local HA application reduces postoperative pain, although effects on trismus and edema remain inconclusive due to heterogeneity and risk of bias across trials—highlighting both promise and the need for better-designed RCTs (Randomized Controlled Trials) [77].

Emerging randomized and controlled clinical data suggest that 0.8–1% HA gels can improve early soft-tissue healing indices and patient-reported outcomes in oral surgery and periodontology. For instance, a split-mouth clinical study found that adjunctive 0.8% HA gel with minimally invasive periodontal surgery improved clinical and radiographic parameters compared with surgery alone, supporting a role for HA in modulating early inflammation and matrix organization [78].

Consistent with HA’s pro-healing and osteoconductive adjunctive effects, a prospective controlled experiment found that 0.8% HA gel improved soft-tissue closure and bone density during follow-up in third-molar sockets [79].

The use of a topical gel containing chitosan after oral surgery improves postoperative comfort and re-epithelialization, and it also reduces analgesic consumption compared to control preparations. These findings are in line with chitosan’s intrinsic muco-adhesion, antimicrobial action, and hemostatic properties [80].

Thermosensitive poloxamer gels loaded with non-steroidal anti-inflammatory drugs (NSAIDs) or local anesthetics provide prolonged analgesia and reduced systemic exposure [81]. For instance, an ibuprofen-loaded mixed micellar gel composed of Poloxamer 403/407 exhibited gelation at ~32 °C and sustained diffusion-mediated drug release, confirming its applicability for mucosal delivery [82].

Additionally, gels containing natural bio-actives (e.g., aloe vera, curcumin, or propolis) have gained attention as adjuncts in mucosal healing due to their antioxidant and antimicrobial activity [83]. The Pluronic F127-based curcumin-loaded in situ gel is a prime example; it has effectively clinically treated periodontitis, has suitable gelation at oral temperatures, and has favorable mucoadhesive characteristics [84].

An emerging application of gel-based systems is their use as coating materials for dental implants. Hydrogels and bioactive gel coatings can be applied to titanium or zirconia implant surfaces to provide localized therapeutic release at the bone–implant interface, particularly during early healing or in cases prone to peri-implant inflammation. These coatings can be loaded with antimicrobials, anti-inflammatory agents, growth factors, or osteogenic ions, enabling sustained release directly at the implant surface without systemic drug exposure.

Additionally, polymeric hydrogel layers can enhance corrosion resistance and reduce ion release from metallic implants under acidic or inflammatory oral conditions. Recent experimental and preclinical studies have demonstrated that chitosan-, hyaluronic acid-, and gelatin-based hydrogel coatings improve early osseointegration, reduce peri-implant microbial colonization, and modulate local immune response, thereby supporting long-term implant stability.

#### 2.6.4. Regenerative and Tissue-Engineering Approaches

In the context of periodontal and bone regeneration, gel-based systems act as scaffolds facilitating cell attachment, proliferation, and differentiation. Injectable hydrogels incorporating growth factors (PDGF, TGF-β, BMP-2) or stem cells derived from the periodontal ligament or dental pulp have demonstrated enhanced osteogenesis and angiogenesis in preclinical models [85]. For instance, PDGF-BB-loaded hydrogels containing periodontal ligament stem cells (PDLSCs) achieved sustained growth factor release, improved cell viability, and enhanced periodontal tissue regeneration in preclinical models [86]. Similarly, BMP-2-loaded hydrogel scaffolds showed superior osteoinductive potential and significantly improved new bone formation in rat calvaria and periodontal defect models [87].

A study by Khayat et al. demonstrated that human dental pulp stem cells (hDPSCs) encapsulated within gelatin–methacryloyl (GelMA) hydrogels exhibited excellent viability, proliferation, and differentiation potential, supporting the formation of vascularized and mineralized tissue in vitro and in vivo [88]. The GelMA hydrogel provided a biomimetic microenvironment that promoted angiogenic marker expression and osteogenic differentiation confirming its dual potential for dental pulp and alveolar bone tissue engineering [88].

Beyond simple hydrogel scaffolds, the integration of gel matrices with advanced 3D-printed or electrospun nanofiber constructs has expanded their therapeutic potential toward fully personalized tissue-engineered systems. A recent study developed a hierarchical 3D-printed bilayer membrane composed of a nanofibrous upper layer and a hydrogel-based lower layer, achieving controlled dual-drug release and accelerated alveolar bone regeneration in a rabbit periodontal defect model [89]. Such a hybrid architecture combines the mechanical stability of solid scaffolds with the biomimetic functionality of hydrogels, resulting in enhanced cell infiltration, vascularization, and mineralized tissue formation.

Furthermore, nanocomposite hydrogels incorporating bioactive nanoparticles such as hydroxyapatite, bioactive glass, or silica-based nanocarriers have demonstrated synergistic effects by providing mechanical reinforcement while releasing osteogenic ions or growth factors [90]. These multifunctional systems allow precise tuning of degradation kinetics and spatial drug delivery, promoting guided tissue regeneration in complex periodontal environments [91], as can be seen in Table 2 below:

In recent years, the evolution of local delivery systems has led to the development of multiple formulations designed to optimize drug stability, bioavailability, and therapeutic response. Among these, gel-based materials represent only one category within a broader spectrum of technologies applied in oral and periodontal therapy.

## 3. Future Directions

Despite considerable progress in the development of gel-based delivery systems for oral applications, several factors continue to limit their clinical translation. Polymeric matrices’ inherent heterogeneity, variations in cross-linking density, salivary flow, and enzymatic degradation all impact drug diffusion profiles, which in turn affects therapeutic effectiveness, which can be quite unpredictable.

Clinical trials are still varied in terms of design, sample size, and duration of follow-up, which makes it hard to compare results or develop standardized clinical indications; most research that are available is preclinical.

From a materials perspective, biocompatibility and mechanical stability remain major challenges, particularly in dynamic environments such as the gingival sulcus or mucosal surfaces. Achieving optimal viscoelasticity, adhesive strength, and degradation kinetics without compromising drug release remains an unresolved objective. Furthermore, sterilization, shelf life, and batch reproducibility represent critical technical barriers to the large-scale manufacturing and regulatory approval of these products [97].

Periodontal and mucosal tissues are biologically complicated, which further limits the potential of delivery systems. These tissues must be able to adjust to changing pH, temperature, and inflammatory conditions [98]. The in vivo performance of thermosensitive and stimuli-responsive gels still varies greatly based on formulation parameters and local microenvironmental conditions, even if new technologies have helped to some extent to overcome these obstacles. Incorporating bioactive compounds, growth factors, or stem cells into a formulation adds another layer of complexity while simultaneously raising questions about stability and safety [99,100].

The integration of nanotechnology, 3D bioprinting, and bio-responsive polymers into hybrid and multifunctional systems that improve spatial precision and regeneration capacity should be the primary focus of future research [101].

In order for studies to be comparable to each other, standardized assessment methodologies that incorporate mechanical, microbiological, and histological outcomes are necessary. To connect innovations in the lab with foreseeable chair-side applications, translational initiatives should prioritize long-term biocompatibility, cost-effectiveness, and patient-centered usability [102,103]. To turn present experimental prototypes into dependable therapeutic treatments in contemporary dentistry practice, a multidisciplinary strategy integrating materials science, pharmacology, and clinical knowledge is necessary.

Despite their promising therapeutic performance, smart, responsive and nanotechnology-based gel systems face several translational challenges that limit their clinical adoption. Manufacturing scalability is currently hindered by batch-to-batch variability in polymer cross-linking, nanoparticle loading efficiency, and gelation behavior under physiological conditions. Moreover, long-term physicochemical stability, sterilization compatibility, and controlled degradation in the oral environment remain insufficiently standardized.

From a regulatory standpoint, nanocomposite and stimuli-responsive gels fall under combined device–pharmaceutical classifications, requiring extensive biocompatibility, toxicity, and release-profile data that are not yet consistently provided across studies. Clinical evidence is also fragmented, with limited multicenter randomized trials, short follow-up periods, and heterogeneous patient selection criteria, making it difficult to determine predictable benefit and safety across diverse clinical scenarios. Addressing these gaps will be essential for moving smart gel formulations from laboratory prototypes to routine clinical practice.

In addition to scientific and technical considerations, successful translation of advanced gel systems into routine clinical use requires addressing regulatory standardization, manufacturing scalability, and cost-effectiveness. The integration of nanomaterials, bioactive agents, and stimuli-responsive components often places these systems under combined pharmaceutical–medical device regulatory frameworks, requiring comprehensive safety, biocompatibility, and release-profile validation. Large-scale manufacturing remains challenging due to the need for precise control of polymer structure, crosslinking density, and nanoparticle uniformity, which may affect reproducibility and batch stability. Furthermore, the production of multifunctional gels incorporating growth factors or stem cells significantly increases cost, limiting accessibility and clinical adoption. Therefore, standardized fabrication protocols, streamlined regulatory pathways, and economic feasibility assessments will be essential to enable broad implementation of next-generation gel delivery systems in dentistry.

## 4. Conclusions

Gel-based delivery systems represent a cornerstone in the evolution of modern dental therapeutics, bridging material science with clinical innovation. Their versatility—ranging from hydrogels and mucoadhesive matrices to thermosensitive and nanoparticle-enriched scaffolds—has enabled controlled, localized, and biocompatible drug administration within the oral cavity. These platforms overcome the limitations of conventional formulations by improving drug stability, reducing systemic exposure, and enhancing patient compliance.

Recent progress has extended their role beyond mere drug carriers toward multifunctional constructs capable of supporting tissue regeneration and biomimetic healing. The integration of bioactive molecules, growth factors, and stem cells into injectable or printable gel matrices has demonstrated tangible benefits in periodontal and endodontic regeneration, as well as in postoperative and mucosal wound care.

Even with these advancements, there are still obstacles to translating these findings into ordinary clinical practice, such as variability in the models used, a lack of standardization, and the necessity for long-term safety validation. To develop reproducible, scalable, and patient-centered solutions, future research should bridge the gap between biomaterials science, pharmacology, and clinical dentistry.

The ultimate goal of developing gel-based platforms is to create more precise and less invasive oral drug delivery systems that combine pharmacological accuracy with regeneration capabilities. This will allow for more individualized dental treatment.

## Figures and Tables

**Table 1:** Structural and functional classification of dental gel-based delivery systems.

Criterion	Category	Examples/Features
By Composition	Natural Polymers	Chitosan, alginate, gelatin—biocompatible, biodegradable, bio adhesive
	Synthetic Polymers	Polyvinyl alcohol, Carbopol, poloxamer—stable, tunable viscosity, controlled degradation
By Function	Controlled-release systems	Gradual drug diffusion and sustained therapeutic levels
	Mucoadhesive systems	Improved adhesion and prolonged contact with oral tissues
	Thermosensitive systems	Sol–gel transition triggered by temperature
	Nanoparticle-loaded systems	Enhanced mechanical and pharmacological performance
	Bioactive/regenerative systems	Promote cell adhesion, proliferation and tissue repair

**Table 2:** Gel-based regenerative systems for periodontal and bone tissue engineering.

Type of Gel System	Incorporated Bioactive(s)	Experimental Model/Application	Main Findings	Reference
PDGF-BB–loaded hydrogel with PDLSCs	Platelet-Derived Growth Factor-BB (PDGF-BB) + Periodontal Ligament Stem Cells (PDLSCs)	In vitro and rat periodontal defect model	Sustained PDGF release; enhanced fibroblast proliferation, angiogenesis, and regeneration of cementum and alveolar bone	[92]
BMP-2–releasing hydrogel scaffold	Bone Morphogenetic Protein-2 (BMP-2)	Rat calvarial and periodontal defect models	Superior osteoinductive potential; increased new bone formation and improved structural integrity	[93]
GelMA hydrogel encapsulating hDPSCs	Gelatin–Methacryloyl (GelMA) + Human Dental Pulp Stem Cells (hDPSCs)	In vitro and in vivo (mouse)	Promoted angiogenic (VEGF, CD31) and osteogenic (RUNX2, OCN) gene expression; formation of vascularized mineralized tissue	[93]
3D-printed bilayer membrane with hydrogel	Hydrogel base + electrospun nanofiber top layer + dual drug system	Rabbit periodontal defect model	Controlled dual-drug release; enhanced alveolar bone and ligament regeneration; improved vascularization	[94,95]
Nanocomposite hydrogel scaffold	Hydroxyapatite nanoparticles + bioactive glass + PEG-based hydrogel	Bone defect model	Controlled degradation; ion-mediated osteogenic response; improved mechanical reinforcement	[96]

## Data Availability

No new data were created or analyzed in this study. Data sharing is not applicable to this article.

## Abbreviations

The following abbreviations are used in this manuscript:

PNIPAAm	Poly(N-isopropylacrylamide)
HPMC	Hydroxypropyl Methylcellulose
NPs	Nanoparticles
PLGA	Polylactic-co-glycolic acid
BMP-2	Bone Morphogenetic Protein-2
VEGF	Vascular Endothelial Growth Factor
Ag	Silver
ZnO	Zinc Oxide
TiO_2_	Titanium Dioxide
MMP	Matrix Metalloproteinase
SRP	Scaling and Root Planing
RCTs	Randomized Controlled Trials
PRF	Platelet-Rich Fibrin
PDGF	Platelet-Derived Growth Factor
TGF-β	Transforming Growth Factor Beta
GelMA	Gelatin–Methacrylate
PDGF-BB	Platelet-Derived Growth Factor-BB
PDLSCs	Periodontal Ligament Stem Cells
GelMC	Gelatin–Methacryloyl
hDPSCs	Human Dental Pulp Stem Cells
RGD	Arginine–Glycine–Aspartic acid
MAPK/ERK	Mitogen-Activated Protein Kinase/Extracellular Signal-Regulated Kinase
Wnt/β-catenin	Wingless/INT-1 signaling pathway
